# Structural and advanced imaging in predicting MGMT promoter methylation of primary glioblastoma: a region of interest based analysis

**DOI:** 10.1186/s12885-018-4114-2

**Published:** 2018-02-21

**Authors:** Yu Han, Lin-Feng Yan, Xi-Bin Wang, Ying-Zhi Sun, Xin Zhang, Zhi-Cheng Liu, Hai-Yan Nan, Yu-Chuan Hu, Yang Yang, Jin Zhang, Ying Yu, Qian Sun, Qiang Tian, Bo Hu, Gang Xiao, Wen Wang, Guang-Bin Cui

**Affiliations:** 1 Department of Radiology & Functional and Molecular Imaging Key Lab of Shaanxi Province, Tangdu Hospital, the Military Medical University of PLA Airforce (Fourth Military Medical University), 569 Xinsi Road, Xi’an, 710038 China; 2 Department of Medical Image Diagnosis, Hanzhong Central Hospital, Hanzhong, Shaanxi 723000 China

**Keywords:** Glioblastoma, Image feature, Oxygen 6-methylguanine-DNA methyltransferase (MGMT) promoter, Apparent diffusion coefficient (ADC), 3-diminsional pseudo-continuous arterial spin labeling (3D pCASL) imaging

## Abstract

**Background:**

The methylation status of oxygen 6-methylguanine-DNA methyltransferase (MGMT) promoter has been associated with treatment response in glioblastoma(GBM). Using pre-operative MRI techniques to predict MGMT promoter methylation status remains inconclusive. In this study, we investigated the value of features from structural and advanced imagings in predicting the methylation of MGMT promoter in primary glioblastoma patients.

**Methods:**

Ninety-two pathologically confirmed primary glioblastoma patients underwent preoperative structural MR imagings and the efficacy of structural image features were qualitatively analyzed using Fisher’s exact test. In addition, 77 of the 92 patients underwent additional advanced MRI scans including diffusion-weighted (DWI) and 3-diminsional pseudo-continuous arterial spin labeling (3D pCASL) imaging. Apparent diffusion coefficient (ADC) and relative cerebral blood flow (rCBF) values within the manually drawn region-of-interest (ROI) were calculated and compared using independent sample *t* test for their efficacies in predicting MGMT promoter methylation. Receiver operating characteristic curve (ROC) analysis was used to investigate the predicting efficacy with the area under the curve (AUC) and cross validations. Multiple-variable logistic regression model was employed to evaluate the predicting performance of multiple variables.

**Results:**

MGMT promoter methylation was associated with tumor location and necrosis (*P* <  0.05). Significantly increased ADC value (*P* <  0.001) and decreased rCBF (*P* <  0.001) were associated with MGMT promoter methylation in primary glioblastoma. The ADC achieved the better predicting efficacy than rCBF (ADC: AUC, 0.860; sensitivity, 81.1%; specificity, 82.5%; vs rCBF: AUC, 0.835; sensitivity, 75.0%; specificity, 78.4%; *P* = 0.032). The combination of tumor location, necrosis, ADC and rCBF resulted in the highest AUC of 0.914.

**Conclusion:**

ADC and rCBF are promising imaging biomarkers in clinical routine to predict the MGMT promoter methylation in primary glioblastoma patients.

## Background

Glioblastoma (GBM) [1], the most common primary malignant brain tumor, accounts for 45% of all malignant primary central nervous system (CNS) tumors and arises most commonly de novo (primary GBM) [2]. Despite multimodal treatments including invasive surgery followed by conformal radiation and chemotherapy, the patients’ median survival remains poor, ranging from 12 to 15 months [3, 4]. The clinical outcome of GBM patients depends on many factors, including age at diagnosis, Karnofsky score, tumor resection extent, the histological classification, tumor grade and genetic alterations of key molecules.

Recently, with the development of molecular pathology, the IDH1/2 gene mutations and oxygen 6-methylguanine-DNA methyltransferase (MGMT) promoter methylation are increasingly used as prognostic or predictive biomarkers for gliomas [5]. IDH status plays an important role in predicting patients’ survival, however, its mutation occurs in only 6% of GBM patients, leaving the vast majority of GBMs be IDH1/2 wild-type. Therefore, IDH mutation could not fully explain the GBM heterogeneity. MGMT inhibits the repair of temozolomide (TMZ)-induced therapeutic DNA damage, and ultimately correlates with progress-free survival (PFS) and overall survival (OS) of GBM patients [6]. Besides, the clinical practice offered evidence that MGMT promoter methylation plays important role in determining therapeutic strategies [7] and associates with better treatment response to TMZ [8]. Thus, it is very important to preoperatively identify the methylation status of MGMT promoter in GBM patients.

The gold standard to identify genetic alterations in GBM is surgical sampling, an necessary invasive procedure for GBM treatment or identification, however, may induce severe complications. Furthermore, GBM heterogeneity and sampling errors increase the risk of erroneous genetic profiling. In contrast, MRI, as a noninvasive method, appears to be an alternative to determine GBM MGMT promoter methylation.

To date, conventional structural image features, including tumor location, tumor volume, enhancement, invasiveness, and edema, have been utilized to predict the MGMT promoter methylation, however without expert consensus [9–11]. Recently, image texture analysis [12] and machine learning [13] based on these conventional MRI are gaining more attention, while the time-consuming methodology is not suitable for routine clinical work. Advanced MRI including dynamic susceptibility contrast (DSC) perfusion imaging [14–17], diffusion-weighted (DWI) [18–20] or diffusion tensor (DTI) imaging [17], arterial spin labeling (ASL) imaging [21] and CT imaging [17], are employed to predict MGMT promoter methylation with unsatisfying accuracies. However, both IDH mutant and wild-type GBMs were included in these studies [9–11, 14–22], without taking the varied image features or survivals between these two populations into consideration. The association between image parameters and MGMT promoter methylation is not clear, especially in IDH-wild-type GBM population.

Thus, we restricted the current analysis in GBM patients with wild-type IDH. The purpose of our study was to seek certain variables derived from conventional structural image features including multifocal, tumor cross midline, tumor location, enhancement, cyst, necrosis, edema, side, which may reflect MGMT promoter methylation status. Meanwhile, we evaluated the efficacy of ADC and rCBF values in predicting MGMT promoter methylation.

## Methods

### Patient population

One hundred and five patients with pathologically confirmed primary GBM from July 2014 to September 2015 were retrospectively investigated in the current study. All patients underwent near total or gross total resection. Patients were included according to the following criteria: (a) confirmative information of MGMT promoter methylation status, (b) the image quality was satisfying without susceptibility or motion artifacts, (c) receiving no corticosteroid when MRI was performed, and (d) presence of solid tumor components available for ADC analysis.

**Fig. 1:**
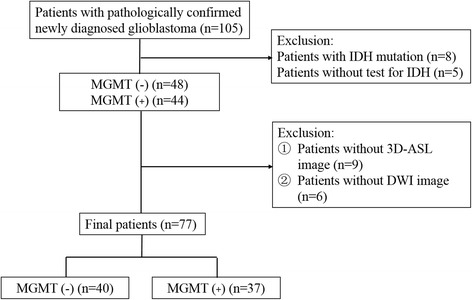
Flow diagram for the patients selection

Five patients without IDH information and 8 with IDH mutation were excluded, leaving 92 enrolled patients in the current study. Nine patients without 3D pCASL and 6 without DWI were excluded. The final study population contained 77 patients (36 men and 41 women; mean age, 55 years; age, 21–87 years) with newly diagnosed and pathologically confirmed GBM with wild-type IDH. The process flow diagram is shown in Fig. 1.

### MRI data acquisition

The whole brain MRI examinations were performed on a 3 T MRI system (Discovery MR750, General Electric Medical System, Milwaukee, WI, USA) with an eight-channel head coil (General Electric Medical System). Conventional MRI, contrast-enhanced MRI, DWI and 3D pCASL imaging were implemented during the examination.

Conventional MRI sequences included axial T_1_w image (TR/TE, 1750 ms/24 ms; matrix size, 256 × 256; FOV, 24 × 24 cm; number of excitation, 1; slice thickness, 5 mm; gap, 1.5 mm), axial T_2_w image (TR/TE, 4247 ms/93 ms; matrix size, 512 × 512; FOV, 24 × 24 cm; number of excitation, 1; slice thickness, 5 mm; gap, 1.5 mm) and sagittal T_2_w image (TR/TE, 10,639 ms/96 ms; matrix size, 384 × 384; FOV, 24 × 24 cm; number of excitation, 2; slice thickness, 5 mm; gap, 1.0 mm), and axial fluid-attenuated inversion recovery (FLAIR) (TR/TE, 8000 ms/165 ms; matrix size, 256 × 256; FOV, 24 × 24 cm; number of excitation, 1; slice thickness, 5 mm; gap, 1.5 mm). DWI was obtained using a single-shot, echo-planar sequence in the axial plane (TR/TE, 3000 ms/minimum ms; FOV, 24 × 24 cm; matrix size, 160 × 160; number of excitation, 2; slice thickness, 5 mm; gap, 1.5 mm). Two b values (0 and 1000 s/mm^2^) were used in three orthogonal directions. The acquisition time for DWI was 24 s. 3D pCASL was acquired prior to the injection of contrast agents, using a 3D spiral fast spin echo (FSE) sequence (TR/TE, 4590 ms/10.5 ms; FOV, 24 × 24 cm; slice thickness, 4 mm; slice number, 40; number of excitation, 3; and post-labeling delay time, 1525 ms).

Finally, contrast-enhanced T_1_-weighted spin echo sequence was acquired in the transverse, sagittal, and coronal planes after intravenous administration of 0.1 mmol/kg gadodiamide (Omniscan; GE Healthcare, Co. Cork, Ireland). 3D T_1_-weighted sequences were also acquired (TR/TE, 8.2 ms/3.2 ms; TI, 450 ms; flip angle 12°; section thickness, 1.2 mm; FOV, 512 × 512 mm; matrix size, 256 × 256; number of excitations, 1; image number, 140).

### MRI data processing and image analysis

All data were transferred to a workstation (Advantage Workstation 4.6, General Electric Medical System, Milwaukee, Wisconsin, USA) for processing.

The conventional structural image features of all patients were assessed independently by two experienced neuroradiologists (Y.-C.H. and L.-F.Y. who have 12 and 6 years of experience, respectively, in neuro-oncology imaging) who were blinded to the MGMT promoter methylation status. A third senior neuroradiologist (G.-B.C, 25 years of experience in brain tumor diagnosis) re-examined the images and determined the final classification of image feature when inconsistency existed between the two neuroradologists. Tumor features derived from the MRI were characterized based on the criteria outlined in Table 1.

**Table 1:** Image definition

Variable	Classification criteria
Location	
Type I	Tumor in which the contrast-enhancing lesion contacts both the SVZ and the cortex
Type II	Tumor contacts the SVZ but not the cortex
Type III	Tumor contacts the cortex but not the SVZ
Type IV	Tumor contacts neither the SVZ nor the cortex
Tumor cross midline	
No	Tumor is limited to the unilateral cerebral hemisphere
Yes	Tumor crosses the brain midline and extends into contralateral cerebral hemisphere
Multifocal	
No	Only one region of tumor, either enhancing or non-enhancing
Yes	At least a region of tumor, either enhancing or non-enhancing, which is not contiguous with the major tumor mass
Necrosis	
No	No necrosis within the tumor
Mild	Necrosis affecting ≤50% of the tumor
Severe	Necrosis affecting > 50% of the tumor
Cyst	
No	No cyst in the tumor
Yes	Cyst in the tumor
Edema	
No	No convincing edema
Mild	Edema extending ≤2 cm from tumor margin
Severe	Edema extending > 2 cm from tumor margin
Enhancement	
Nodular	Largest focal contrast-enhancement diameter of tumor ≤1.5 cm
Patchy	Maximum contrast-enhancement diameter of tumor>1.5 cm
Ringlike	Cystic necrosis with peripheral enhancement
Side	
Left	Tumor located in left cerebral hemisphere
Right	Tumor located in right cerebral hemisphere

Note: Location classification was based on the spatial relationship of the contrast-enhancing lesion to the subventricular zone. (SVZ = subventricular zone)

First, the two radiologists independently reviewed the conventional plain and contrast-enhanced MR images carefully to determine the solid part of tumor before ADC and CBF analyses. Next, a region of interest (ROI) was manually drawn on the solid part of the tumor with relatively higher signal on DWI and lower ADC value in ADC map. Similarly, ROI was drawn to include the solid elements of tumor with relatively higher signal on CBF map by using 3D contrast enhanced T_1_weighted imaging(3D–T1WI/C) as cross-reference. The mean ROI area of the lesions was 55.7 ± 6.3 mm^2^ (range, 41.0–73.0 mm^2^). Meanwhile, for both the ADC and 3D pCASL analyses, hemorrhagic, calcified, cystic, necrotic areas and large vessels were avoided. Each radiologist made three ROIs on the lesion side, and the mean value was calculated as the final value. To conduct the normalization, CBF value on the lesion side was normalized to that on the contralateral side with same size ROI: rCBF = CBF value on the lesion side/CBF value on the contralateral side.

### Statistical analysis

All statistical analyses were performed by using SPSS 20.0 software (SPSS Inc., Chicago, IL, USA) and WEKA software (WEKA version 3.8.1). A *P* value < 0.05 was considered to indicate a statistical significance.

*Analyses of conventional structural image features*. - Categorical data of conventional structural image features (location, tumor cross midline, multifocal, necrosis, edema, cyst, enhancement and side) between MGMT promoter methylated group and unmethylated group were analyzed using the Fisher’s exact test.

*Diagnostic performance with ADC or rCBF*. - Quantitative data (ADC value and rCBF) were denoted as the mean and standard deviation. The normal distribution of data was investigated with Kolmogorov-Smirnov (K-S) test. The differences of ADC and rCBF values between MGMT promoter methylated and unmethylated groups were analyzed by using independent sample *t* test. ROC analyses were performed to determine the efficacies (sensitivity, specificity, and AUC) of different parameters in predicting MGMT promoter methylation. The *Youden* index was employed to identify the optimal cut-off value. Additionally, leave-one-out cross-validation (LOOCV) was used to prevent over-fitting, and validated AUC was obtained as well.

*Efficacies of imaging parameter combinations.* - A multivariate logistic regression model (LRM) with LOOCV was used to investigate the efficacies of image parameter combinations in predicting MGMT promoter methylation.

*Analysis of agreement*. - The intra-class correlation coefficient (ICC) analysis was used to assess the inter-reader agreement on measuring image parameters. The ICC was interpreted as poor (< 0.4), moderate (≥ 0.4 but < 0.75), and good (> 0.75).

## Results

### Differences in conventional structural image features between MGMT promoter methylated and unmethylated groups

The conventional structural image features of the 92 GBM patients were summarized in Table 2. Tumor location was significantly different between MGMT promoter methylated and unmethylated groups (*P* = 0.012), implying that the subventricular zone (SVZ) was more likely to be spared in patients with MGMT promoter methylation. Besides, MGMT promoter methylation is prone to be associated with tumor necrosis (*P* = 0.028). Other qualitative image features were not significantly different between these two groups, including multifocal (*P* = 0.114), tumor cross midline (*P* = 0.478), cyst (*P* = 0.335), edema (*P* = 0.688), enhancement (*P* = 0.259) and side (*P* = 0.720).

**Table 2:** Correlations between MGMT status and image features

	Unmethylated	Methylated	Total	*P*-value*
Location, n (%)				0.012
Type I	12/48 (25)	12/44 (27)	24/92 (26)	
Type II	16/48 (33)	4/44 (9)	20/92 (22)	
Type III	18/48 (39)	20/ 44 (45)	38/92 (41)	
Type IV	2/48 (4)	8 /44 (18)	10/92 (11)	
Tumor cross midline, n (%)				0.478
No	38/48 (79)	36/44 (82)	74/92 (80)	
Yes	10/48 (21)	8/44 (18)	18/92 (20)	
Multifocal, n (%)				0.114
No	39/48 (81)	30/44 (68)	69/92 (75)	
Yes	9/48 (19)	14/44 (32)	23/92 (25)	
Necrosis, n (%)				0.028
No	2/48 (4)	0	2/92 (2)	
Mild	26/48 (54)	15/44 (34)	41/92 (45)	
Severe	20/48 (42)	29/44 (66)	49/92 (53)	
Cyst, n (%)				0.335
No	40/48 (83)	39/44 (87)	79/92 (86)	
Yes	8/48 (17)	5/44 (13)	13/92 (14)	
Edema, n (%)				0.688
No	10/48 (21)	6/44 (14)	16/92 (17)	
Mild	25/48 (52)	26/44 (59)	51/92 (55)	
Severe	13/48 (27)	12/44 (27)	25/92 (28)	
Enhancement, n (%)				0.259
Nodular	11/48 (23)	9/44 (20)	20/92 (22)	
Patchy	7/48 (15)	2/44 (5)	9/92 (10)	
Ringlike	30/48 (62)	33/44 (75)	63/92 (68)	
Side, n (%)				0.720
Left	20/48 (42)	19/44 (43)	39/92 (42)	
Right	22/48 (46)	17/44 (39)	39/92 (42)	
Midline	6/48 (12)	8/44 (18)	14/92 (16)	

Note: The *P* values* were calculated from the Fisher’s exact test

### Performance of single advanced MRI parameter (ADC and rCBF) in predicting MGMT promoter methylation

**Fig. 2:**
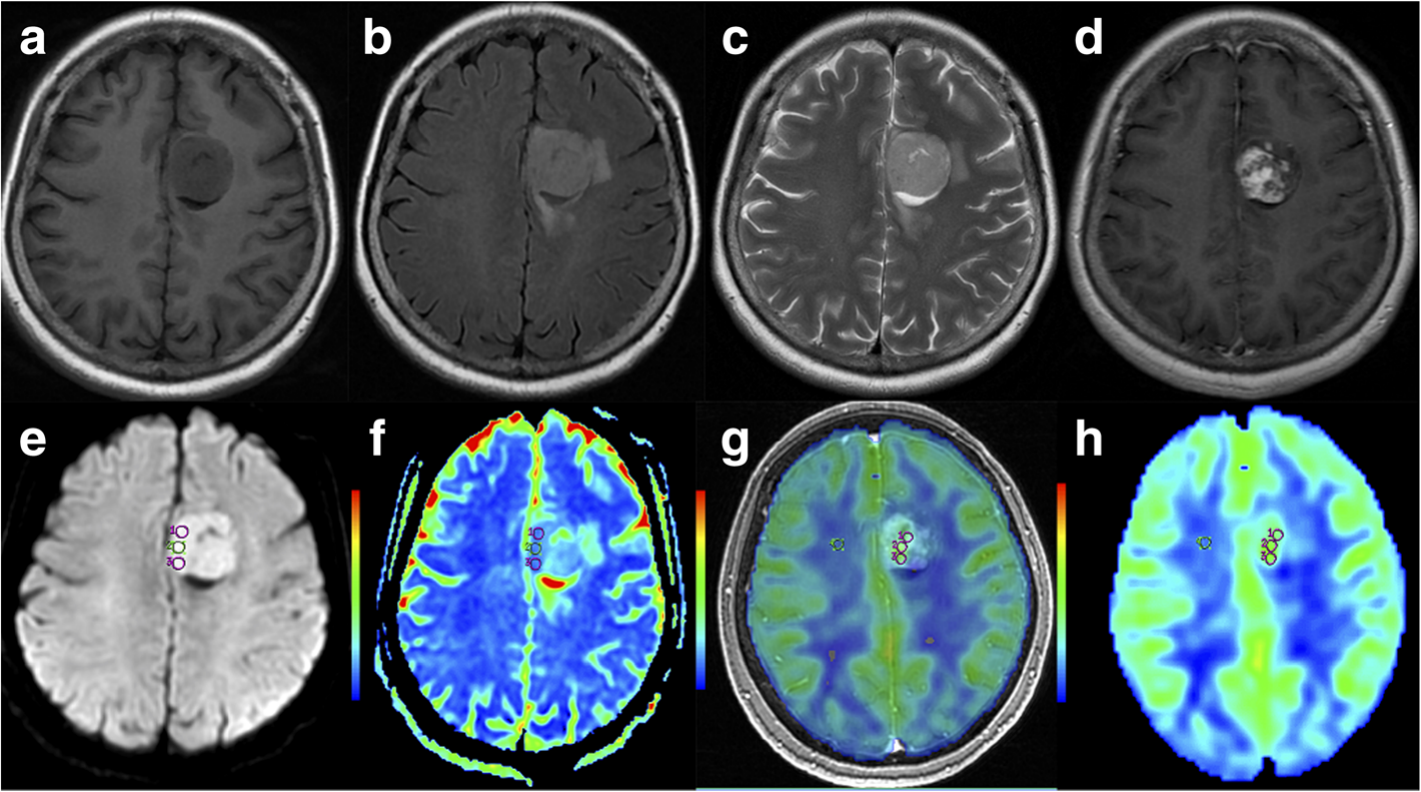
Structural (**a, b, c** and **d**) and advanced (**e, f, g** and **h**) MR images obtained from a 53-year-old female showing left frontal lobe GBM with MGMT promoter methylation. **a** Precontrast T_1_-weighted image. **b** Fluid-attenuated inversion recovery image. **c** T_2_-weighted image. **d** Postcontrast T_1_-weighted image. **e** DWI map. **f** ADC map: the mean ADC value of the three small ROI was 0.722 × 10^− 3^ mm^2^/s. **g** The map fused CBF and 3D–T1WI/C. **h** CBF map: the mean rCBF of the three small ROI was 3.94

**Fig. 3:**
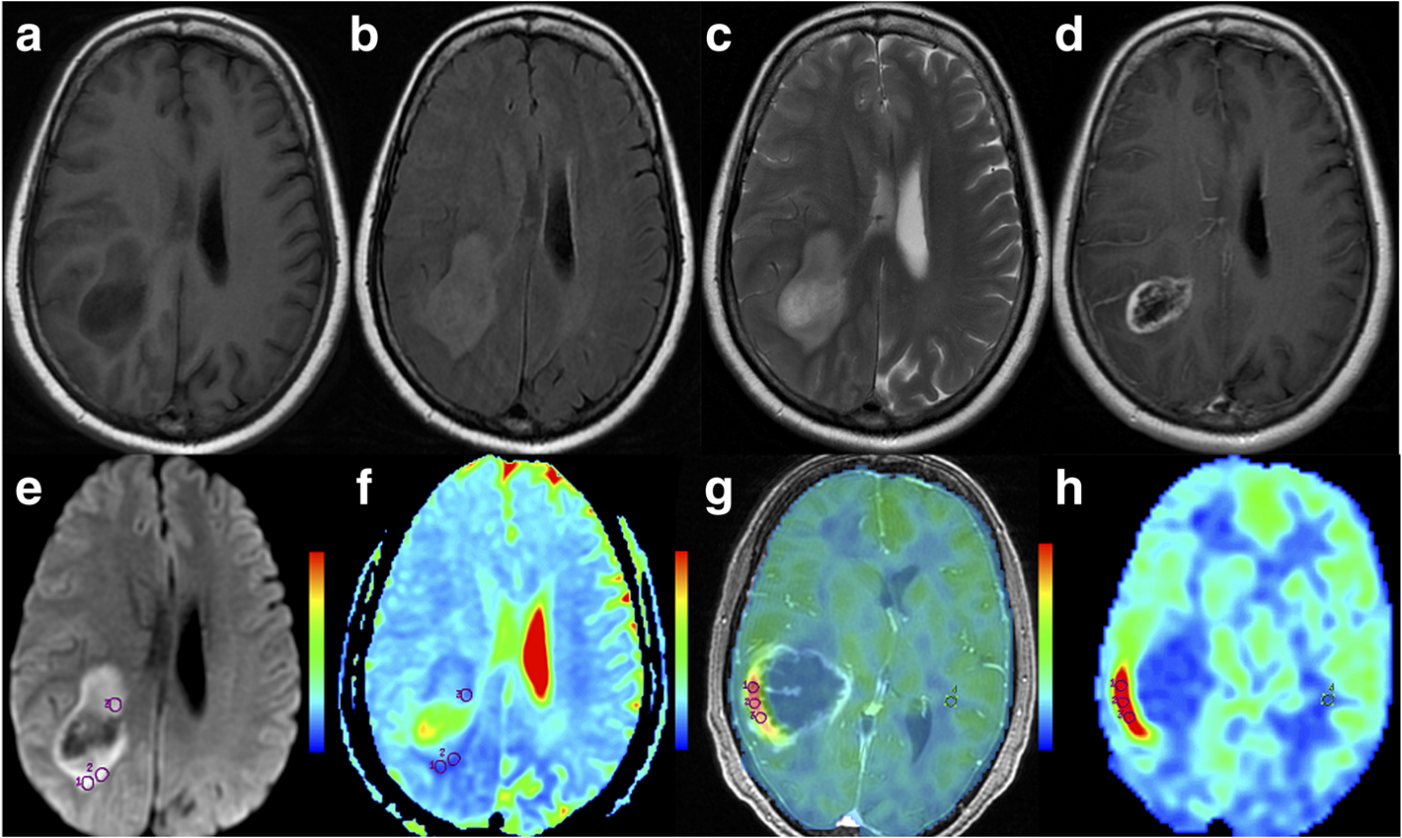
Structural (**a**, **b**, **c** and **d**) and advanced (**e**, **f**, **g** and **h**) MR images obtained from a 55-year-old male showing right temporal and parietal lobe GBM without MGMT promoter methylation. **a** Precontrast T1-weighted image. **b** Fluid-attenuated inversion recovery image. **c** T2-weighted image. **d** Postcontrast T1-weighted image. **e** DWI map. **f** ADC map: the mean ADC value of the three small ROI was 0.661 × 10^− 3^ mm^2^/s. **g** The map fused CBF and 3D–T1WI/C. **h** CBF map: the mean rCBF of the three small ROI was 10.64

The descriptive statistics of the DWI and 3D pCASL parameters between MGMT promoter methylated and unmethylated groups were shown in Table 3. The rCBF of GBMs with unmethylated MGMT promoter (9.467 ± 2.706, *n* = 40) was significantly higher than that of the methylated group (5.916 ± 2.518, *n* = 37) (*P* <  0.001). The ADC value was lower in MGMT promoter unmethylated group (0.729 ± 0.085 × 10^− 3^ mm^2^/s, n = 40) than that of the methylated group (0.889 ± 0.137 × 10^− 3^ mm^2^/s, n = 37) (*P* <  0.001). The representative cases were shown in Figs. 2 and 3**.**

**Table 3:** Differences in ADC and rCBF values between MGMT (−) and MGMT (**+**) ($\overline{x}$±s)

Values	MGMT(−)	MGMT(+)	t	*P*-value*
rCBF	9.467 ± 2.706	5.916 ± 2.518	5.945	< 0.001
ADC (×10^−3^ mm^2^/s)	0.729 ± 0.085	0.899 ± 0.137	6.514	< 0.001

Note: ADC = apparent diffusion coefficient, rCBF = relative cerebral blood flow, MGMT (−) = MGMT promoter unmethylated, MGMT (+) = MGMT promoter methylated

The *P* values* were calculated from the independent sample *t* test

In predicting MGMT promoter methylation, the AUC of the ADC (0.860) was higher than that of the rCBF (0.835). The optimal thresholds for predicting MGMT promoter methylation were 0.792 × 10^− 3^ mm^2^/s for ADC and 7.680 for rCBF (Table 4). By using LOOCV, the cross-validated AUC and accuracy were 0.842 and 79.2% for ADC and 0.814 and 74.0% for rCBF, respectively (Table 4).

**Table 4:** ROC and LOOCV of ADC and rCBF values for differentiating MGMT (+) from MGMT (−)

Values	AUC	Sensitivity (%)	Specificity (%)	Cutoff value	LOOCV Accuracy(%)	LOOCV AUC
rCBF	0.835	75.0	78.4	7.680	74.0	0.814
ADC (×10^−3^ mm^2^/s)	0.860	81.1	82.5	0.792	79.2	0.842

Note: ADC = apparent diffusion coefficient, rCBF = relative cerebral blood flow, AUC = area under ROC curve, LOOCV = leave-one-out cross-validation, MGMT (−) = MGMT promoter unmethylated, MGMT (+) = MGMT promoter methylated

### Efficacies of parameter combinations for predicting MGMT promoter methylation

On ROC analyses, the AUC for predicting MGMT promoter methylation was the highest when location, necrosis, ADC and rCBF (0.914) were combined, and the lowest when location and necrosis (0.670) were combined (Table 5). For differentiating MGMT promoter methylation from unmethylation, the addition of ADC or rCBF to the combination of location and necrosis significantly improved the AUC from 0.670 to 0.891 (when ADC was added) and from 0.670 to 0.852 (when rCBF was added), respectively.

**Table 5:** Comparison of AUC of the varied MRI parameter combinations

Values	AUC	Sensitivity (%)	Specificity (%)	LOOCV Accuracy (%)	LOOCV AUC
rCBF + ADC	0.893	83.1	83.2	81.8	0.871
Location + Necrosis	0.670	62.3	62.3	53.2	0.597
rCBF + Location + Necrosis	0.852	77.9	77.6	72.7	0.821
ADC + Location + Necrosis	0.891	80.5	80.2	79.2	0.845
ALL	0.914	85.7	85.2	80.5	0.877

Note: ADC = apparent diffusion coefficient, rCBF = relative cerebral blood flow, AUC = area under ROC curve, LOOCV = leave-one-out cross-validation, ALL = ADC + rCBF + Location + Necrosis

Compared to combination of location, necrosis and ADC/rCBF, LOOCV offered evidence that the combination of ADC and rCBF showed the higher diagnostic accuracy for distinguishing MGMT promoter methylation from unmethylation. Compared with combination of ADC and rCBF, the addition of location and necrosis did not significantly improved cross-validated AUC. The cross-validated accuracy were 81.8% (for the combination of ADC and rCBF) and 80.5% (for the combination of location, necrosis, ADC and rCBF) (Table 5). The results of the ROC analyses and cross-validations for the combined imaging parameters were summarized in Table 5, respectively.

### Analysis of agreement

As shown in Table 6, the inter-observer ICC value for ADC and rCBF was close to 1 (*P* <  0.001), suggesting excellent measurement reliability of quantitative MRI parameters.

**Table 6:** Intraclass Correlation Coefficient between the two readers

Values	ICC	*P*-value*	95% CI
rCBF	0.921	< 0.001	0.875–0.950
ADC (×10^−3^ mm^2^/s)	0.911	< 0.001	0.860–0.943

Note: P values were calculated from intraclass correlation coefficient with the two-way random model

## Discussion

In the present study, we revealed that the methylation status of the MGMT promoter in IDH-wild-type GBM was associated with conventional structural image features (tumor location and necrosis). Moreover, the diagnostic performance of ADC was found to be slightly better than that of rCBF in predicting MGMT promoter methylation. In addition, the integrative performance of combined structural image features (location and necrosis) and quantitative parameters (ADC and rCBF) achieved the highest AUC, compared to the combination of location and necrosis or ADC/rCBF.

In the current study, among all the investigated conventional structural image features, only tumor location and necrosis were correlated with MGMT promoter methylation status. First, GBMs with methylated MGMT promoter were more frequently associated with severe necrosis. This result is inconsistent with previous report [23]. One possible explanation may be that methylation of the MGMT promoter decreases the MGMT protein level. As a DNA repair enzyme, decreased MGMT led to severe DNA damage and cell death, eventually more necrosis in tumor. Second, our result demonstrated that SVZ was more likely to be spared in patients with MGMT promoter methylation. Previous studies suggested that MGMT promoter methylation is associated with GBM in parietal and occipital lobes [23], the left hemisphere and temporal lobe [10] or independent from tumor location [13]. To our knowledge, this is the first report showing the relationship between involvement of SVZ and MGMT promoter methylation in GBMs population with wild-type IDH. It is now generally accepted that tumor location, as an important image feature [24] associated with genetic features, is closely related with patient prognosis [25]. Our findings may help explain why MGMT promoter methylated GBMs, which spare the SVZ, have prolonged survival. However, since type I-II contacting the SVZ may be skewed in large tumors, and is not necessarily the same as the SVZ arising ones with the center in the SVZ, cautions should be paid when dealing with such cases and further studies are needed to clarify this issue.

ADC [25, 26] was used as a potential surrogate biomarker for MGMT promoter methylation, however, with controversies [15–20, 22, 27, 28]. In our study, we revealed that the ADC value in GBMs with MGMT promoter methylation was higher than in those without MGMT promoter methylation. In accordance with our results, several previous studies showed that ADC ratios or ADC minimum values were lower in tumors with unmethylated MGMT promoters than with methylated promoters [17, 18] and that mean ADC had a positive relationship with the MGMT promoter methylation ratio [22]. However, lower ADC value in MGMT promoter methylated GBMs was reported in a recent histogram analysis study [27]. Besides, no significant correlation between ADC values and MGMT promoter methylation status was also reported [20, 28]. These conflicting results may be partially attributed to different methodologies, including ROI selection (only contrast enhanced portion of the tumor), and different cohorts (high-grade glioma, versus GBMs only). This study is different from previous ones in the study population (IDH-wild-type GBMs only versus both IDH mutant and IDH-wild-type GBMs [15–20, 28]) and ROI (ADC minimum area of tumor solid part, not simply contrast enhanced portion of the tumor [29]), which may explain the difference between ours and previous ones. Further investigation with a larger cohort and unified protocol is needed.

3D pCASL, providing valuable information about tumor angiogenesis, can be an important biological marker for tumor grading [30] and treatment response predicting [31]. However, to our knowledge, no significant correlation between rCBF and MGMT promoter methylation was reported in only one study [21]. However, a major confounding factor in that study is the fact that both IDH mutant and IDH-wild-type GBMs were included, with a relatively small number of patients in each group for MGMT promoter methylation analysis. Here, significant differences in the rCBF between MGMT promoter methylated and unmethylated GBMs was confirmed in the current study. A recent study [32] showed a direct link between MGMT expression and decreased angiogenesis of GBM cells, with the unclear mechanism. Therefore, the rCBF provided by 3D pCASL may be a potential image parameter to predict the MGMT promoter methylation. Taking together, both ADC and rCBF performed well in their standing alone ability to predict MGMT promoter methylation in this study. It is generally accepted that varied functional MR techniques can provide different valuable information about tumor microenvironment, which makes the combinational utilization of multimodal MRIs necessary. Our study indeed demonstrated that combination of ADC and rCBF improved the performance in predicting MGMT promoter methylation over single modality parameter. Adding advanced modality parameters (ADC or rCBF) to conventional ones (tumor location and necrosis) further improved the diagnostic accuracy. Given that neither rCBF nor ADC requires an exogenous contrast agent, our results imply that ADC and rCBF is especially useful as an adjunct for predicting MGMT promoter methylation when the patients’ condition is not appropriate for contrast agent administration.

There are some limitations for the current study. First, the sample size was relatively small. Second, analysis of patients’ overall survival and the match of surgical specimen with the corresponding image were not performed. Third, although the measurement consistency between the two independent radiologists was good, however, possible bias still existed due to the manual drawing and/or visually positioning ROIs.

## Conclusions

The methylation status of the MGMT promoter in IDH-wild-type GBM was associated with conventional structural image features (tumor location and necrosis). ADC peroformed slightly better than rCBF in predicting MGMT promoter methylation. Combining structural image features (location and necrosis) and quantitative parameters (ADC and rCBF) achieved the higher AUC.

## Abbreviations

3DpCASL: 3-diminsional pseudo-continuous arterial spin labeling
ADC: Apparent diffusion coefficient
ASL: Arterial spin labeling
CBF: Cerebral blood flow
CNS: Central nervous system
DSC: Dynamic susceptibility contrast
DTI: Diffusion tensor imaging
DWI: Diffusion-weighted imaging
FLAIR: Fluid-attenuated inversion recovery
GBM: Glioblastoma
ICC: Intra-class correlation coefficien
LOOCV: Leave-one-out cross-validation
LRM: Logistic regression model
MGMT: Oxygen 6-methylguanine-DNA methyltransferase
PFS: Progress-free survival
ROI: Region of interest
SVZ: Subventricular zone
TMZ: Temozolomide
